# Child stunting concurrent with wasting or being overweight: A 6-y follow up of a randomized maternal education trial in Uganda

**DOI:** 10.1016/j.nut.2021.111281

**Published:** 2021-04-16

**Authors:** Per O. Iversen, Moses Ngari, Ane C. Westerberg, Grace Muhoozi, Prudence Atukunda

**Affiliations:** a Department of Nutrition, Institute of Basic Medical Sciences, University of Oslo, Oslo, Norway; b Department of Hematology, Oslo University Hospital, Oslo, Norway; c The Childhood Acute Illness & Nutrition Network, Nairobi, Kenya; d KEMRI/Wellcome Trust Research Programme, Kilifi, Kenya; e Institute of Health Sciences, Kristiania University College, Oslo, Norway; f Department of Human Nutrition and Home Economics, Kyambogo University, Kampala, Uganda

**Keywords:** Anthropometry, Children, Maternal education, Overweight, Randomized trial, Stunting, Uganda, Wasting

## Abstract

**Objectives:**

There is paucity of longitudinal data on combined anthropometric deficiencies in children. Herein, we present data on child stunting concurrent with wasting or being overweight among children in a 6-y follow-up study of a maternal education trial in rural Uganda.

**Methods:**

We previously performed a randomized controlled trial where half of 511 mothers of 6- to 8-mo children were given a 6-mo education concerning nutrition, hygiene, and child stimulation. Anthropometry and prevalence of stunting with wasting or being overweight were determined. We applied multilevel mixed-effect logistic regression models and *χ*^2^ statistic to assess the effects of the intervention and trend in prevalence over time, respectively.

**Results:**

Complete data sets were obtained from 307 of 511 children (60%). The prevalence of stunting and wasting or being overweight was <7% both, and did not change significantly over time. Notably, the prevalence of concurrent stunting and being overweight was significantly reduced in the intervention group compared with the controls among children age 36 mo and 60 to 72 mo, with corresponding odds ratios at 0.24 (95% confidence interval, 0.06−0.90) and 0.10 (95% confidence interval, 0.01−0.82), respectively.

**Conclusions:**

The prevalence of stunting concurrent with wasting or being overweight remained low during the observation period. The intervention may have reduced concurrent stunting and being overweight over time.

## Introduction

The progress toward achieving United Nations Sustainability Development Goal 2 (zero hunger) has stagnated or reversed in sub-Saharan Africa [1]. Adding to this burden of undernutrition is the increase in children being overweight [2]. In support of this, the Food and Agriculture Organization recently presented dismal data on child nutritional status. Among children age <5 y, 144 million (21.3%) were stunted (low height for age, which is a marker of chronic undernutrition), 47 million (9%) were wasted (low weight for height, which is a marker of acute malnutrition), and approximately 38 million (5.6%) were overweight in 2019 [3].

Stunting, wasting, and being overweight are associated with chronic diseases. Children concomitantly stunted and wasted or being overweight have a high risk of later morbidity and mortality [4]. However, there is a lack of longitudinal studies on this double burden of malnutrition, which has also not been examined in randomized controlled trials (RCTs) emphasizing educational interventions.

To prevent stunting, we previously performed a cluster RCT examining the effects of maternal education to Ugandan mothers [5]. We now performed a long-term follow-up study of this RCT, and herein report data on stunting concurrent with wasting or being overweight among these children during their first 6 y.

## Methods

We randomized (1:1) 511 mother−child pairs to an intervention or control group when the children were age 6 to 8 mo. The maternal education intervention emphasized nutrition, hygiene, and child stimulation [5], and is detailed in the supplementary material. The children were assessed at age 6 to 8 mo, 12 to 16 mo, 20 to 24 mo, 36 mo, and 60 to 72 mo. Approvals were obtained from the Uganda National Council for Science and ClinicalTrials.gov (ID NCT 02098031).

Anthropometry was measured according to World Health Organization guidelines, and stunting and wasting were defined as z-scores <−2 standard deviation (SD) from the median of a reference population [6]. For children age >59 mo, wasting was defined using body mass index for age z-score <−2 SD. Overweight was defined as weight for height z-score >2 SD for children age <60 mo and body mass index for age z-score >1 SD for those age ≥60 mo [7]. Multilevel mixed-effect logistic regression models with a cluster as the random intercept were used with binary stunting, wasting, being overweight, concurrent stunting and wasting or being overweight as the dependent variable and the randomized arm as the independent variable. The ^2^ statistic was used to assess the effects of the intervention and trend in prevalence over time. Statistical significance was set at *P* < 0.05.

## Results

A total of 307 of 511 children recruited at age 6 to 8 mo (60%) had complete anthropometry data. Among the 511 children, three died in the intervention and three in the control group. In addition, 94 and 104 children in the intervention and control group, respectively, had missing values (did not attend visits or had relocated). All baseline characteristics were balanced between the children randomized to the intervention and control groups and not significantly different from those in the original RCT, suggesting that the follow-up sample was representative of the original trial cohort (data not shown).

In the intervention group, the prevalence of stunted children increased to 58% at age 20 to 24 mo before declining to 30% at age 60 to 72 mo. The corresponding values among the controls were a 63% increase and 31% decline, respectively (Table 1). The prevalence of wasting was rare with no wasting at age 20 to 24 mo. Being overweight was more common at age 36 mo, affecting 23% and 21% of children randomized to the intervention and control groups, respectively. We did not detect any significant difference in the prevalence of stunted, wasted, and overweight children between the two study groups at any timepoint.

Concurrent stunting and wasting was rare (Table 1). At age 6 to 8 mo, only 1.8% of children randomized to the intervention group were concurrently stunted and wasted, but none were among the controls. At age 12 to 16 mo, the corresponding prevalence remained at 1.8% in the intervention group and 5.0% among the controls (P = 0.14). Notably, at age 20 to 24 mo and 60 to 72 mo, no child was concurrently stunted and wasted, but at 36 mo, 0% and 0.7% of children were concurrently stunted and wasted in the intervention and control groups, respectively.

Only 3.0% and 4.3% of children in the intervention and control group, respectively, were concurrently stunted and overweight at age 6 to 8 mo (Table 1). At age 12 to 16 mo, only one intervention child was concurrently stunted and overweight, but 3.6% and 0.7% of children in the intervention and control groups, respectively, were concurrently stunted and overweight at age 20 to 24 mo (P = 0.13). Notably, at age 36 mo, more children randomized to the control group (7.1%) than to the intervention group (1.8%) were concurrently stunted and overweight (P = 0.03). Similarly, at age 60 to 72 mo, more children in the control (5.7%) than in the intervention (0.6%) group were concurrently stunted and overweight (P = 0.03). There was no time trend in the prevalence of children concurrently stunted and overweight (P = 0.28 for intervention group; P = 0.43 for controls).

## Discussion

The prevalence of stunting was markedly higher than that for wasted and overweight children. The coexistence of stunting and wasting or being overweight was low and with no time dependency. The combination of stunting and wasting was <5%, and supported a recent meta-analysis of children age 6 to 59 mo from 84 countries with a pooled prevalence of 3% [8]. Owing to the low prevalence, we could not provide a reliable estimate of changes in concurrent stunting and wasting over time. Despite this, efforts should be made to reduce concurrent stunting and wasting because of increased mortality risk.

The prevalence of concurrent stunting and being overweight was low and similar to a large survey conducted among schoolaged children in 57 low- and middle-income countries [9]. Interestingly, we found that the intervention significantly reduced the prevalence of concurrent stunting and being overweight at age 36 mo and 60 to 72 mo compared with controls. Intriguingly, our intervention initiated with children age 6 to 8 mo can possibly prevent this combined malady 2 to 4 y later.

The main strength and novelty of our study are the multiple data collections from the start of complementary feeding to school age. The study also originates from a robustly designed trial. Study limitations include the low participant number and a lack of data on dietary intakes and body composition.

## Conclusions

The prevalence of child stunting with concurrent wasting or being overweight was low and did not change during this 6-y follow-up study. Our maternal education may have reduced the prevalence of concurrent stunting and being overweight over time. More research of the mechanisms governing combined anthropometrical deficits is needed to identify effective treatment.

## Supplementary Material

Supplementary

## Figures and Tables

**Table 1:** Longitudinal anthropometry data from enrollment into randomized controlled trial until children reached school age

Anthropometrical measure	Intervention, n (%) (n = 166)	Control, n (%) (n = 141)	Odds ratio (95% confidence interval)*	P-value
Stunting				
6−8 mo	34 (20)	40 (28)	0.65 (0.38−1.10)	0.11
12−16 mo	69 (42)	51 (36)	1.26 (0.79−1.99)	0.34
20−24 mo	96 (58)	89 (63)	0.79 (0.43−1.45)	0.45
36 mo	85 (51)	85 (60)	0.68 (0.40−1.15)	0.15
60−72 mo	49 (30)	43 (31)	0.95 (0.52−1.74)	0.86
Wasting				
6−8 mo	8 (4.8)	5 (3.6)	1.38 (0.44−1.31)	0.58
12−16 mo	14 (8.4)	13 (9.2)	0.96 (0.34−2.75)	0.94
20−24 mo	0	0	_*	_
36 mo	0	2 (1.4)	_*	_
60−72 mo	0	1 (0.7)	_*	_
Overweight				
6−8 mo	11 (6.6)	10 (7.1)	0.93 (0.33−2.65)	0.89
12−16 mo	7 (4.2)	8 (5.7)	0.73 (0.26−2.07)	0.26
20−24 mo	2 (1.2)	1 (0.7)	1.69 (0.09−30.6)	0.72
36 mo	39 (23)	29 (21)	1.19 (0.61−2.34)	0.61
60−72 mo	11 (6.6)	17 (12)	0.52 (0.23−1.15)	0.10
Concurrent stunted and wasted				
6−8 mo	3 (1.8)	0	_†	_
12−16 mo	3 (1.8)	7 (5.0)	0.35 (0.09−1.40)	0.14
20−24 mo	0	0	_†	_
36 mo	0	1 (0.7)	_†	_
60−72 mo	0	0	_†	_
Concurrent stunted and overweight				
6−8 mo	5 (3.0)	6 (4.3)	0.70 (0.21−2.34)	0.56
12−16 mo	1 (0.6)	0	_†	_
20−24 mo	6 (3.6)	1 (0.7)	5.25 (0.62−44.1)	0.13
36 mo	3 (1.8)	10 (7.1)	0.24 (0.06−0.90)	0.03
60−72 mo	1 (0.6)	8 (5.7)	0.10 (0.01−0.82)	0.03

We focused on stunting, wasting, and being overweight as categorical variables defined by the World Health Organization, because our emphasis was not on continuous estimates of growth development.

* Odds ratios and *P*-values are from the multilevel logistic regression models.

† Not enough values to calculate odds ratios.
